# An F-Actin Mega-Cable Is Associated With the Migration of the Sperm Nucleus During the Fertilization of the Polarity-Inverted Central Cell of *Agave inaequidens*

**DOI:** 10.3389/fpls.2021.774098

**Published:** 2021-11-24

**Authors:** Alejandra G. González-Gutiérrez, Antonia Gutiérrez-Mora, Jorge Verdín, Benjamín Rodríguez-Garay

**Affiliations:** ^1^Unidad de Biotecnología Vegetal, CIATEJ, Centro de Investigación y Asistencia en Tecnología y Diseño del Estado de Jalisco, A.C., Zapopan, Mexico; ^2^Unidad de Biotecnología Industrial, CIATEJ, Centro de Investigación y Asistencia en Tecnología y Diseño del Estado de Jalisco, A.C., Zapopan, Mexico

**Keywords:** Polygonum-type embryo sac, chalazal central cell polarity, cytoplasmic strands, double fertilization, helobial endosperm, Asparagaceae

## Abstract

Asparagaceae’s large embryo sacs display a central cell nucleus polarized toward the chalaza, which means the sperm nucleus that fuses with it during double fertilization migrates an atypical long distance before karyogamy. Because of the size and inverted polarity of the central cell in Asparagaceae, we hypothesize that the second fertilization process is supported by an F-actin machinery different from the short-range F-actin structures observed in *Arabidopsis* and other plant models. Here, we analyzed the F-actin dynamics of *Agave inaequidens*, a classical Asparagaceae, before, during, and after the central cell fertilization. Several parallel F-actin cables, spanning from the central cell nucleus to the micropylar pole, and enclosing the vacuole, were observed. As fertilization progressed, a thick F-actin mega-cable traversing the vacuole appeared, connecting the central cell nucleus with the micropylar pole near the egg cell. This mega-cable wrapped the sperm nucleus in transit to fuse with the central cell nucleus. Once karyogamy finished, and the endosperm started to develop, the mega-cable disassembled, but new F-actin structures formed. These observations suggest that Asparagaceae, and probably other plant species with similar embryo sacs, evolved an F-actin machinery specifically adapted to support the migration of the fertilizing sperm nucleus within a large-sized and polarity-inverted central cell.

## Introduction

Two sperm cells are released from the pollen tube at the egg apparatus boundary during Angiosperm’s fertilization. One of the sperm fuses the egg cell leading to the first plasmogamy and, subsequently, the first karyogamy that generates the zygote (Hamamura et al., 2011). Almost simultaneously, the second sperm fuses with the central cell leading to the second plasmogamy and karyogamy and further endosperm development (Berger et al., 2008). In *Arabidopsis*, whose central cell nucleus is polarized toward the micropylar end (Sprunck and Gross-Hardt, 2011), the distance the second sperm nucleus travels from the plasmogamy site to the central cell nucleus is around 1 μm (Kawashima and Berger, 2015). However, species in the Asparagaceae family, along with other 13 monocotyledonous families, harbor embryo sacs with a polarity-inverted central cell nucleus, i.e., it localizes near the chalazal pole (Davis, 1966; Bhojwani and Bhatnagar, 1983). In *Agave tequilana*, the distance between the egg cell and the central cell nucleus is about 200-times longer than in *Arabidopsis* (González-Gutiérrez et al., 2014). The latter implies that the second sperm nucleus needs to undertake a longer journey in Asparagaceae. Thus, it is plausible that these plant species evolved a specialized long-range transport machinery to support the migration of the sperm nucleus.

An established model to explain the sperm nuclei migration during fertilization proposes they are carried to the fusion sites by a cytoskeleton-supported mechanism (Huang and Russell, 1994; Zhang and Russell, 1999; Wallwork and Sedgley, 2000; Ye et al., 2002). Kawashima et al. (2014) demonstrated that F-actin, but not microtubules, transports the immotile sperm nuclei during *Arabidopsis* fertilization. Once inside the central cell, the second sperm nucleus is surrounded by an aster-shaped F-actin structure (Kawashima et al., 2014) that migrates in synchrony with inward moving (plasma membrane to central cell nucleus) F-actin cables attached to the plasma membrane by formins and ROP8 (Ali et al., 2020; Ali and Kawashima, 2021). These F-actin cables form a mesh-like structure whose movement and stability depend on class XI myosin XI-G (Ali et al., 2020). A similar F-actin arrangement and meshwork movement have been observed in rice zygotes (Ohnishi and Okamoto, 2017), which suggested the migration mechanism of this sperm nucleus might be general among angiosperms (Ali et al., 2020). Nevertheless, a physical connection between the aster-shaped structure and the inward moving F-actin cables has not been established yet.

The F-actin arrangement for cargo transport has been demonstrated to largely rely on the distance the cargo has to travel. Precise short-distance cargo exocytosis is usually mediated by fanned thin actin cables arrays (Geitmann and Emons, 2000). On the other hand, thick actin cables are primarily associated with sizeable long-distance movements, such as those that allow the movement of organelles in root hairs and pollen tubes (Chebli et al., 2013). Nevertheless, the precise mechanisms that determine the different conformations that actin can adopt remain unknown (Geitmann and Emons, 2000).

Because of the exceptional configuration of the embryo sac of Asparagaceae species and the F-actin arrangement dependence on the distance the cargo needs to travel, we hypothesize F-actin-supported migration of the second sperm nucleus in Asparagaceae central cell fertilization may be different from that observed in *Arabidopsis* and other classical plant models. As this megagametophyte configuration is found beyond Asparagales, a similar mechanism for long-distance migration of the sperm nucleus may be more widespread. Here, we addressed such hypotheses by characterizing F-actin structures of the *Agave inaequidens* megagametophyte, from the mature embryo sac and sperm nuclear migration during double fertilization to the early endosperm development.

## Materials and Methods

### Plant Material

Inflorescences with mature flowers from *A. inaequidens* plants were collected in the State of Jalisco, Mexico, during the flowering seasons (May-June) of 2017–2020.

### Pollination and Collection of Specimens

Inflorescences were maintained at the laboratory in freshwater. Flowers were emasculated at anthesis and covered with glassine paper bags to avoid free pollination. Extracted anthers were kept in Petri dishes at 4°C until dehiscence. At this time, mature pollen grains were recovered from the anthers and tested for viability using the *in vitro* method for pollen germination of the *Agave* genus, described by López-Díaz and Rodríguez-Garay (2008).

Once the stigmas of emasculated flowers were receptive (presence of a pollination drop), ten flowers per inflorescence were selected and processed as described below. Unpollinated flowers collected at this stage were considered “time 0.” The remaining flowers at this developmental stage were hand-pollinated (cross-pollination) and collected at different times between 1 and 48 h after pollination (HAP). Thus, it was possible to record actin cytoskeleton dynamics in mature *A. inaequidens* embryo sacs during double fertilization and the first endosperm divisions (Table 1). Ovules of the same flower were dissected with fine-point tweezers and an insulin needle under the stereoscope and evenly divided into two centrifuge tubes to be processed using the histological techniques described below.

**TABLE 1:** Fertilization timing and F-actin dynamics in *Agave inaequidens.*

**Hours after pollination (HAP)**	**F-actin structural change before, during and after fertilization**	**Sample number with reported F-actin structures**
Time 0–18^*^	Mature embryo sac-actin filaments are restricted to perinuclear and cortical areas of each cell of the four cellular types.	*n* = 40
24–30	Actin filaments at the central cell micropylar and chalazal ends started to project from the cortical area to the center of the cell. An arch-shaped F-actin accumulation is formed in the vicinity of the egg apparatus.	*n* = 43
3–36	Parallel F-actin cables extending from the central cell nucleus and aligned to the chalazal-micropylar axis, formed the actin-tunnel.	*n* = 50
38–42	F-actin mega-cable connected the nucleus with the micropylar end of the central cell, close to the egg cell. The sperm nucleus was observed at different stages of its journey (half-, 3/4 and close to the central cell nucleus, just before karyogamy).	*n* = 81
44–48	Early stages of endosperm development, from first division of the primary endosperm nucleus to the eight nucleate stage. F-actin is located around each endosperm nuclei and connects them to each other.	*n* = 50

*These patterns were observed in embryo sacs from non-pollinated (time 0) and pollinated flowers up to 18 HAP.

Only “normal” megagametophytes were considered, i.e., piriform embryo sacs with a pronounced haustorial tube and the four cellular types contained in seven cells. Collapsed embryo sacs and embryo sacs lacking any cellular type due to abnormal growth of the nucellar tissue were discarded. At least 500 ovules encompassing the different developmental stages were analyzed with each staining technique.

### Feulgen Staining

Although Feulgen staining (Barrell and Grossniklaus, 2005) primarily binds to DNA (Kalinowska et al., 2020), some other structures, such as cell walls (Barrell and Grossniklaus, 2005) and cytoplasm can be weakly stained (Chieco and Derenzini, 1999). Feulgen staining adapted with minor modifications for agave ovules was used as the primary method for analyzing the general development stage of embryo sacs. In short, after fixation in FAA (10:5:50:35 formaldehyde: acetic acid: ethanol: distilled water) for 24 h and kept overnight in 70% ethanol, 4°C, ovules were treated with 1 M HCl for 1.5 h, 5.8 M HCl for 2 h, and 1 M HCl for 1 h at room temperature. Subsequently, ovules were rinsed three times with distilled water and stained with Schiff reagent (Sigma cat. no. S5133) for 3 h at room temperature. Dehydration was carried out by an increasing concentration series of 30, 50, 70, 90, 95, 100% ethanol for 30 min, and an additional 100% ethanol incubation for 30 min. Finally, ovules were clarified by a series of methyl salicylate: ethanol solutions of 3:1, 1:1, 1:3 for 1 h each. For observation, samples were mounted in 100% methyl salicylate and examined on a Leica TCS SPE confocal microscope at Ex = 532 nm and Em = 555–700 nm. Images were acquired and processed with the LAS X^®^ software (Leica Microsystems).

### F-Actin Whole-Mount Staining

F-actin whole-mount staining was performed as reported by González-Gutiérrez et al., 2020. Ovules previously dissected were pre-incubated in ASB (Actin Stabilizing Buffer) (50 mM PIPES, 10 mM EGTA, and 1 mM MgCl2, pH 6.8 adjusted with 10 M KOH) at 55°C for 5 min. Then, ovules were fixed with 4% formaldehyde in ASB for 10 min at room temperature (25°C). Afterward, ovules were washed twice with ASB. Two quick rinses with acetone (−20°C), followed by a 5 min incubation in acetone (−20°C), were performed for cuticle solubilization and membrane permeabilization. After this time elapsed, acetone was removed from the microtubes, and ovules were washed 3 times with ASB until the solution remained crystalline. Ovules were then incubated in blocking solution (1% BSA in ASB) for 20 min at room temperature and stained with 0.33 μM rhodamine-phalloidin and 3 μg/ml Hoechst 33258 (diluted in blocking solution), overnight at 4°C. Before clarification, ovules were dehydrated in an increasing concentration series of isopropanol (diluted in ASB) at 4°C, for 7 min each: 75, 85, 95, 100%, and an additional 100% isopropanol step for 12 min. Tissue clarification was carried out by adding 1:1 methyl salicylate-isopropanol solution until all ovules precipitated at the microtube bottom. Finally, ovules were incubated in 100% methyl salicylate for at least 30 min before observation. Samples were analyzed under a Leica TCS SPE confocal microscope using a 532 nm laser for rhodamine-phalloidin (ex/em = 540/556 nm) and a 405 nm laser for Hoechst 33258 observation (ex/em = 352/461 nm). Images were taken and managed with the LAS X^®^ software (Leica Microsystems).

## Results

### *Agave inaequidens* Harbors a Central Cell With Inverted Polarity

**FIGURE 1:**
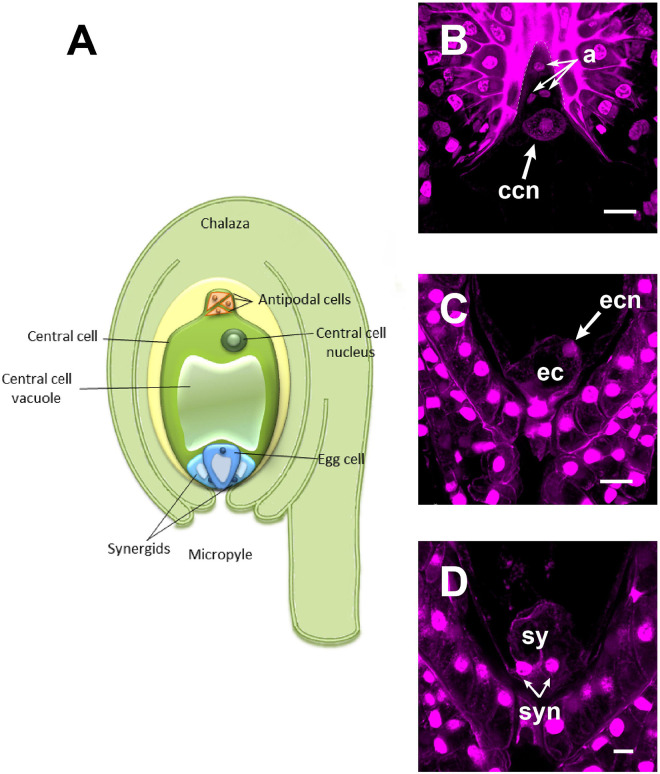
The mature female gametophyte of *Agave inaequidens* (time “0”). **(A)** Scheme of a seven-celled embryo sac arranged in four cell types: three antipodal cells, placed just below the hypostase, and a large central cell with its nucleus polarized toward the chalaza **(B)**. At the micropylar end, the egg apparatus is composed of an egg cell **(C)** and two synergids **(D)**. **(B–D)** Are Feulgen-stained z-stack images. a, antipodal cells; ccn, central cell nucleus; ec, egg cell; ecn, egg cell nucleus; sy, synergids; syn, synergids nuclei. Bar in **(B,C)** = 20 μm and **(D)** = 10 μm.

To elucidate the mechanism that supports the transport of sperm nuclei during the central cell fertilization in Asparagaceae species, we studied the mature embryo sac of non-pollinated flowers of a so far uncharacterized family member: *A. inaequidens. A. inaequidens* mature embryo sac (238.58 ± 16.28 μm, long; and 128.51 ± 12.20 μm, wide; *n* = 40) was piriform with an hypostase at the chalazal end, below which three antipodal cells were located (Figures 1A,B). Moreover, the embryo sac harbored a large central cell (144.03 ± 13.96 μm, long; 124.79 ± 8.89 μm, wide; *n* = 40). Its vacuole occupied most of the central part of the cell, while its nucleus was polarized toward the chalazal pole, just below the antipodal cells (Figures 1A,B). The egg apparatus was located at the opposite side of the embryo sac (central cell nucleus-egg cell nucleus distance: 156.28 ± 22.62 μm, *n* = 40), composed of an egg (Figures 1A,C) and two synergid cells (Figures 1A,D).

### F-Actin in the Mature Embryo Sac Is Restricted to Perinuclear and Cortical Areas

**FIGURE 2:**
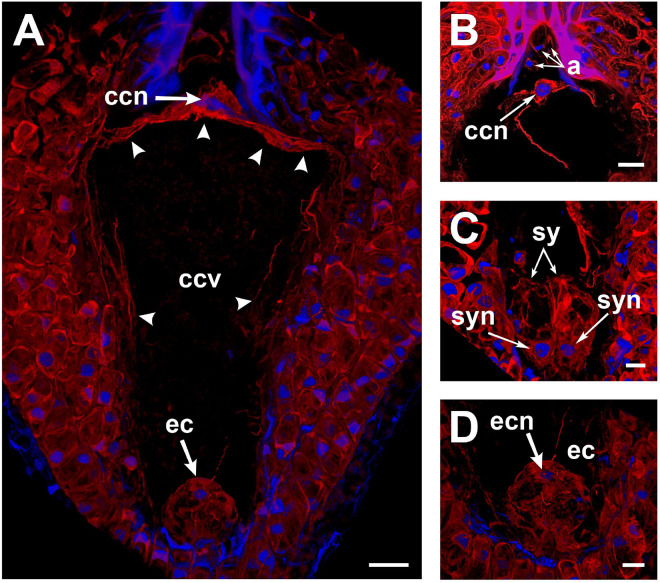
Perinuclear and cortical F-actin in the *Agave inaequidens* mature embryo sac from time “0” up to 18 HAP. **(A)** Rhodamine-phalloidin-stained actin filaments were located at the periphery of the central cell. **(B)** The central cell nucleus displayed a dense perinuclear F-actin coat, while it was less abundant in the antipodal cells. **(C)** F-actin was denser at the synergids’ micropylar end, where their nuclei were located. **(D)** Egg cell cortical and perinuclear actin filaments. ccn, central cell nucleus; ccv, central cell vacuole; a, antipodal cells; sy, synergids; syn, synergid nucleus; ec, egg cell; ecn, egg cell nucleus. Arrow heads, cortical actin filaments of the central cell. All micrographs are z-stack projections oriented with the chalazal pole at the top. Bar in **(A–C)** = 20 μm and **(D)** = 10 μm.

To observe F-actin structures, mature ovules from *A. inaequidens* non-pollinated flowers were stained with rhodamine-phalloidin. Actin filaments were observed layering the cytosolic side of the membrane of each cell of the mature embryo sac of *A. inaequidens* flowers (Figure 2A). The nuclei of these cells were also enveloped by actin filaments that extended until reaching the cell periphery (Figures 2A–D). Compared to profuse actin filaments in the egg apparatus (Figures 2A,C,D), perinuclear actin filaments were less abundant around nuclei of antipodal cells (Figure 2B). In addition to perinuclear actin, synergids displayed a pronounced aggregation of actin filaments at the micropylar end, around the space occupied by their nuclei (Figure 2C). A similarly biased F-actin accumulation, but oriented to the chalazal pole, was observed around the egg cell nucleus (Figure 2D).

In the central cell nucleus, perinuclear F-actin was observed as a dense coat from which several actin filaments extended toward the cell periphery, attaching it to the chalazal area of the cell (Figure 2A). Most of the space in the central cell was occupied by a large vacuole (Figure 2A), and actin filaments were restricted to the cell periphery (Figure 2A).

### F-Actin Cables Projected From the Central Cell Nucleus Form a Tunnel-Like Structure Before Fertilization

**FIGURE 3:**
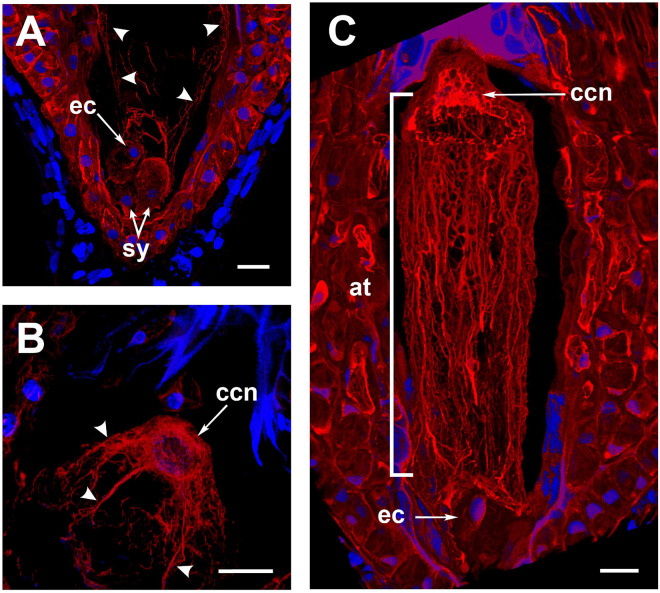
The formation of the *Agave inaequidens* actin-tunnel. **(A)** Central cell F-actin starts accumulating at the cell body. **(B)** Actin filaments projected from the central cell nucleus toward the embryo sac micropylar pole (24–30 HAP). **(C)** Actin-tunnel formed by several parallel filaments extends from the central cell nucleus to the micropylar pole, next to the egg cell (32–36 HAP). ec, egg cell; sy, synergid; ccn, central cell nucleus; at, actin tunnel. Arrowheads in **(A,B)** = actin filaments. In all cases, F-actin was stained with rhodamine-phalloidin. Nuclei were stained with Hoechst 33258. All micrographs are z-stack projections oriented with the chalazal pole at the top. Bar in **(A–C)** = 20 μm.

To observe changes in the actin cytoskeleton during double fertilization, *A. inaequidens* flowers were hand-pollinated and collected at different hours after pollination (HAP, Table 1). In female gametophytes processed between 24 and 30 HAP, actin filaments at the central cell micropylar end started to project from the cortical area to the cell center (Figure 3A); in addition, an arch-shaped accumulation of filaments could be seen close to the egg apparatus (Figure 3A). Simultaneously, the F-actin coat of the central cell nucleus began to extend toward the middle part of the cell, forming thick F-actin cables parallel to the chalazal-micropylar axis (Figure 3B). Finally, around 32–36 HAP, those F-actin cables reached the micropylar end, in the vicinity of the egg apparatus, building a structure we named “actin tunnel,” which generated a lobular chamber (Figure 3C and Supplementary Video 1).

### An F-Actin Mega-Cable Interacts With the Migrating Sperm Nucleus During the Central Cell Fertilization

**FIGURE 4:**
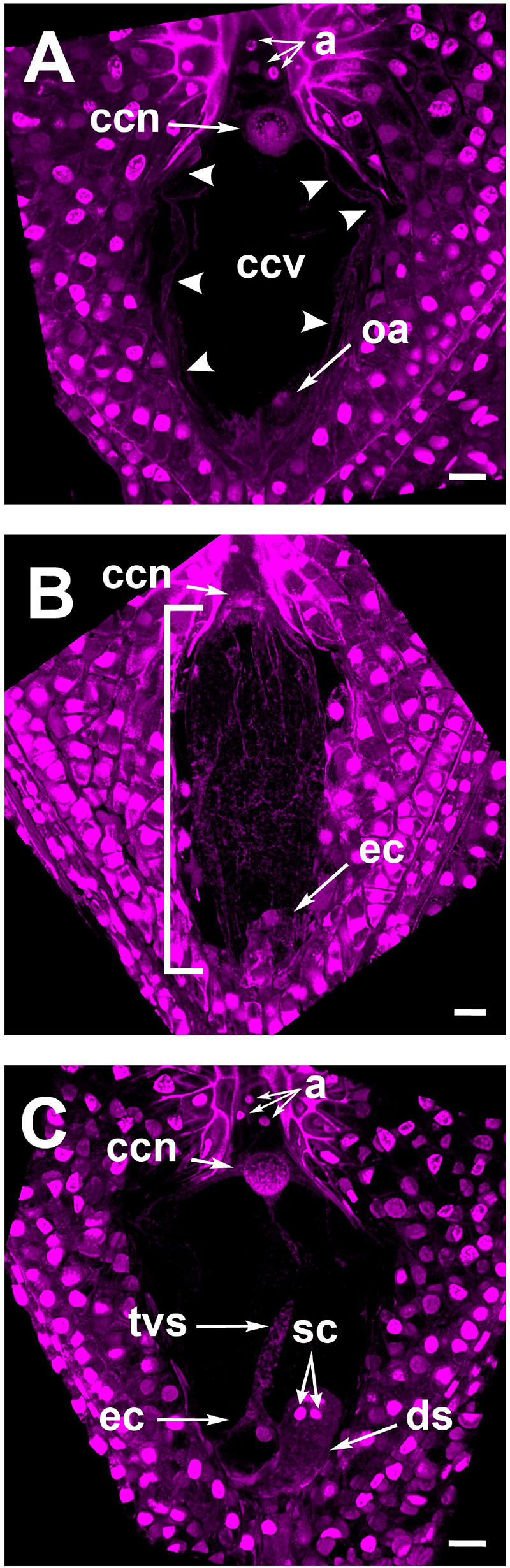
Feulgen stained ovules of *Agave inaequidens* show the developmental specificity of the transvacuolar strand. **(A)** Mature embryo sac at 1–18 HAP. **(B)** Embryo sac at 32–36 HAP. **(C)** Detection of the transvacuolar strand at 38–42 HAP. The two sperm nuclei remain at the chalazal end of the receptive synergid after their discharge but before karyogamy with central and egg cells occurs. a, antipodal cells; ccn, central cell nucleus; ccv, central cell vacuole; oa, ovular apparatus; ec, egg cell; tvs, transvacuolar strand; sc, sperm cells; ds, degenerating synergid. The bracket indicates the accumulation of longitudinal cytoplasmic strands. All micrographs are z-stack projections oriented with the chalazal pole at the top. Bar in **(A–C)** = 20 μm.

**FIGURE 5:**
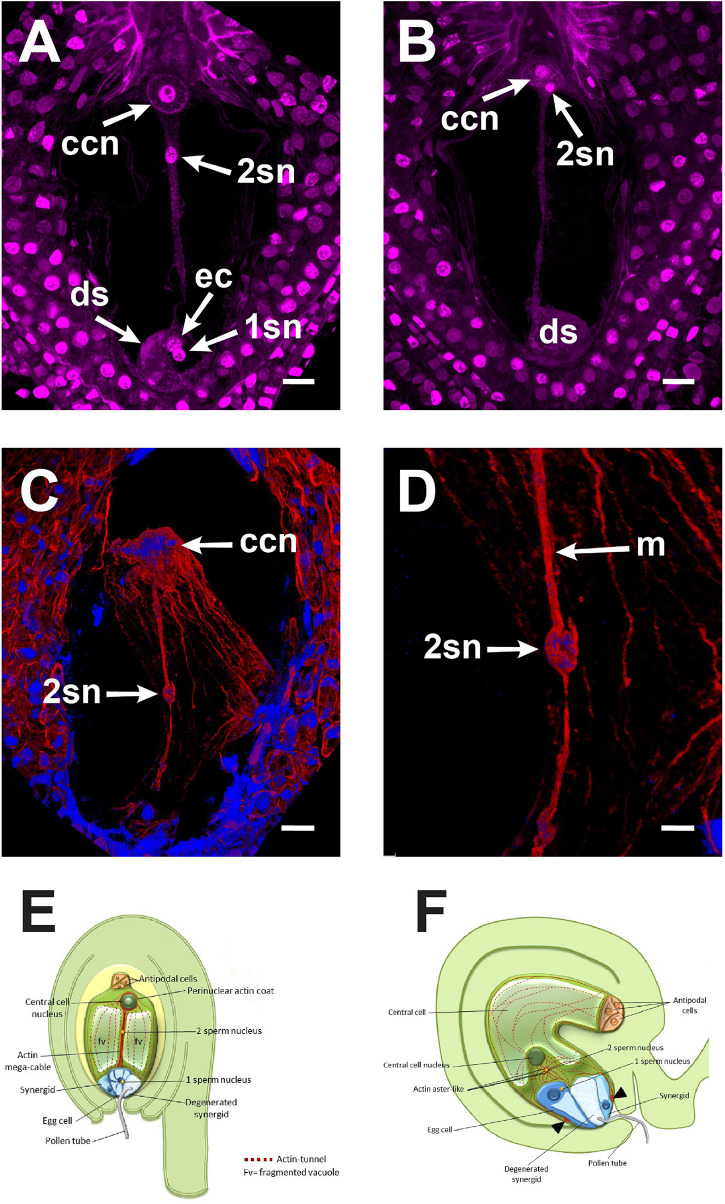
The central cell fertilization of *Agave inaequidens* occurred from 38 to 42 HAP. **(A)** A cytoplasmic trans-vacuolar strand and the second sperm nucleus revealed by Feulgen staining were observed at the central cell. **(B)** Second sperm nucleus getting close to karyogamy. **(C)** The rhodamine-phalloidin stained actin mega-cable traversing the central cell vacuole wrapped the second sperm nucleus (stained with Hoechst 33258). **(D)** Close up of the sperm (in C) wrapped by actin. **(E)** Model of the central cell fertilization in *Agave* embryo sac where an actin-based mega-cable traverses the central vacuole, wraps the sperm nucleus and supports its migration for the second karyogamy event. **(F)** In *Arabidopsis*, during the second fertilization, the sperm nucleus is surrounded by an aster-shaped structure that moves it toward the central cell one (inspired in Dresselhaus et al., 2016). ccn, central cell nucleus, 2sn = second sperm nucleus, ec, egg cell; ds, degenerated synergid; m, actin mega-cable. All micrographs are z-stack projections oriented with the chalazal pole at the top. Bar in **(A–C)** = 20 μm and **(D)** = 10 μm.

As stated in section “F-Actin in the Mature Embryo Sac Is Restricted to Perinuclear and Cortical Areas” for rhodamine-phalloidin staining, Feulgen-stained mature embryo sacs from non-pollinated flowers also showed a large vacuole occupying most of the space in the central cell. This developmental configuration was preserved even in ovules from pollinated flowers up to 18 HAP (Figure 4A); later, cytoplasmic accumulations in the form of thin longitudinal strands started to appear into the central cell (32–36 HAP) (Figure 4B). Subsequently, the pollen tube arrived at the receptive synergid in the embryo sac (38–42 HAP) and released the two sperm cells that moved together toward the chalazal end of the synergid (Figure 4C and Supplementary Video 2). At this stage, a “central strand” traversing the putative central cell vacuole could be observed (Figures 4C, 5A). This trans-vacuolar strand connected the central cell nucleus directly to the micropylar end at the egg cell boundary (Figure 5A). Afterward, plasmogamy and karyogamy of one of the sperm with the egg cell had occurred (Figure 5A). Meanwhile, the second sperm and the central cell fused their membranes, and the nucleus of the former started a journey through the large central cell vacuole moving along the trans-vacuolar strand (Figure 5A) to reach the central cell nucleus at the opposite side of the embryo sac (38–42 HAP) (Figure 5B).

Just before the second fertilization took place, a new thick F-actin cable that we name “mega-cable” began to extend from the actin coat of the central cell nucleus toward the micropylar pole of the cell, more precisely at the boundary of the central cell with the egg cell (Figure 5C). Since it was not possible to catch the early stages of the mega-cable development, it remains unclear whether the mega-cable formed from one or several of the pre-existing tunnel-forming cables or emerged *de novo* as a specialized structure. Moreover, the mega-cable encompassed the DAPI-stained sperm nucleus in transit to the central cell nucleus (Figures 5C,D). Imaging showed that the sperm nucleus moved “inside” the mega-cable rather on it, as may be expected if the nucleus migrates in a myosin-dependent way (Figures 5C,D).

**FIGURE 6:**
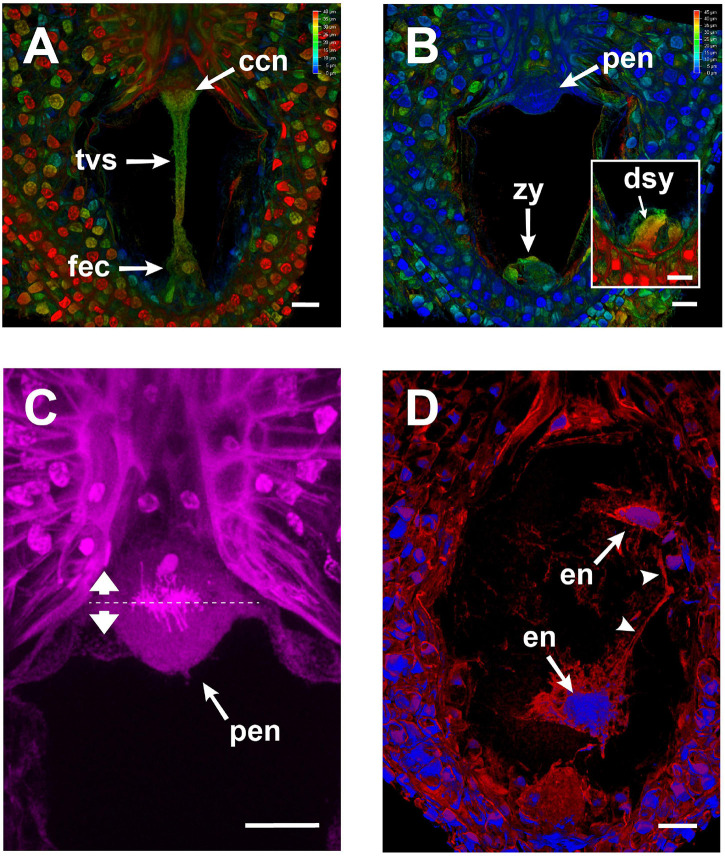
Changes in the embryo sac of *Agave inaequidens* after karyogamy and early endosperm development (44–48 HAP). **(A,B)** Color-coded projection of z-stacked micrographs of similar thickness from Feulgen-stained fertilized ovules. **(A)** A transvacuolar strand connected the chalazal and micropylar poles right after karyogamy of the egg and central cells. **(B)** The transvacuolar strand was no longer detectable before the primary endosperm nucleus divided. **(C)** Close up of the primary endosperm nucleus at anaphase; the dashed line shows the division plane. **(D)** F-actin (red) surrounded and connected (arrowheads) each nucleus (blue) of the developing endosperm in the coenocyte. F-actin was stained with rhodamine-phalloidin; nuclei were stained with Hoechst 33258. ccn, central cell nucleus; tvs, transvacuolar strand; fec, fertilized egg cell; zy, zygote; dsy, degenerated synergid; pen, primary endosperm nucleus; en, endosperm nuclei. Micrographs are z-stack projections oriented with the chalazal pole at the top. Bar in **(A–D)** = 20 μm.

The actin tunnel, the F-actin mega-cable, and the transvacuolar strand were present until karyogamy of the two sperm nuclei with the egg and central cells, respectively, was completed (Figure 6A). Those structures were disassembled just before the primary endosperm nuclei divided for the first time (Figures 6B,C). Subsequently, new F-actin structures around all endosperm nuclei were built, which connected them to each other (Figure 6D).

## Discussion

Enormous progress has been gained in understanding the cellular mechanisms involved in sperm nuclear migration for karyogamy during double fertilization. It is generally accepted that the sperm nuclear migration is an actin-dependent process in both monocot and dicotyledonous species (Kawashima et al., 2014; Ohnishi et al., 2014; Peng et al., 2017). Recent analysis of central cell fertilization in *A. thaliana* shows that an F-actin mesh-like structure which moves from the periphery to the center of the cell, along with an F-actin star-shaped structure that encloses the sperm nucleus, mediates the transit of the latter to the central cell nucleus (Kawashima et al., 2014). Similar processes have been observed in other plant models (Ohnishi and Okamoto, 2017) and the mechanism has been proposed as a general one for Angiosperms (Ali et al., 2020). However, none of the plant species analyzed thus far have possessed a chalazal-polarized central cell nucleus. Members of the Asparagaceae family exhibit this feature, which, together with their large-sized embryo sacs, requires the sperm nucleus to travel an atypical long distance through the central cell in order to achieve karyogamy (González-Gutiérrez et al., 2020). Moreover, it has been observed that the conformation of the F-actin cables largely depends on the distance the cargo needs to travel (Geitmann and Emons, 2000). Because of the latter, we hypothesized that Asparagaceas could support the migration of the sperm nucleus during fertilization of the central cell in a different way to that described for *Arabidopsis*.

In order to test this hypothesis, we characterized the mature embryo sac of non-pollinated flowers from *A. inaequidens*, an agave mainly distributed in temperate areas of Mexico (1400–3000 MASL) (Torres-García et al., 2019). This monocarpic species belongs to the subfamily Agavoideae of the Asparagaceae family, formerly Agavaceae (Angiosperm Phylogeny Group [APG III], 2009). Typically, the flowering stalk is cut off when it starts to grow at 8–10 years and is used as food, or is allowed to accumulate carbohydrates in order to produce alcoholic beverages (Gentry, 1982; Figueredo et al., 2014). It is used in the traditional Mexican beverage industry, but less studied than classical *Agave* species, such as *Agave tequilana*. The mature embryo sac of *A. inaequidens* displays the Polygonum-type with the typical shape and polarization of the central cell nucleus also seen in *A. tequilana, A. colimana* (González-Gutiérrez et al., 2014; Barranco-Guzmán et al., 2019) and other Asparagales (Figure 1). It also shows the classical Asparagales final position of the secondary nucleus in the central cell relative to the micropylar-chalazal axis (Figure 1; Tilton and Lersten, 1981), as well as the presence of an hypostase at the proximal part of the nucellus, close to the antipodal cells (Rudall, 1997).

Polygonum-type megagametophyte is considered the Angiosperm’s ancestral development pattern (Palser, 1975; Haig, 1990). It is present in more than 70% of the Angiosperms (Maheshwari, 1950). In Polygonum-type, the central cell nucleus is generally positioned at the central part of the cell or close to the egg apparatus at the micropylar pole, i.e., like in the classical gametophyte developmental models *A. thaliana* and *Zea mays* (Russell, 1978; Webb and Gunning, 1990). Variations to this developmental pattern have been reported for different plant families and are conserved within them (Bhojwani and Bhatnagar, 1983; Herr, 1984; Tobe, 1989). Variations include the behavior of mature antipodal cells (which can be ephemeral, persist after fertilization, or proliferate) (Tilton, 1978; Williams and Friedman, 2004; Holloway and Friedman, 2008), the timing of the polar nuclei fusion (Jensen, 1973), and the final position of the secondary nucleus in the central cell relative to the micropylar-chalazal axis (Tilton and Lersten, 1981). Embryo sacs with the central cell nuclei polarized toward the chalazal end have been observed in several members of the Asparagaceae family, and in other 17 angiosperm families, 14 of them belonging to the monocotyledoneae class (Davis, 1966).

After confirming the Asparagales-like embryo sac configuration of *A. inaequidens*, the F-actin dynamics along the fertilization of its central cell was characterized. When staining non-pollinated mature embryo sacs with rhodamine-phalloidin, actin filaments were found at each cell’s cortical and perinuclear areas (Figure 2). Actin filaments provide structural stability to the plasma membrane and contribute to the polarization and anchoring of nuclei within a cell (Davidson and Cadot, 2020), which, in turn, are developmentally programmed and associated with the cell function (Smith, 2003; Starr and Han, 2003; Gu et al., 2005). In the central cell of *A. inaequidens*, cortical actin filaments were restricted to the cell periphery, alongside the plasma membrane, while most of the space was occupied by its large vacuole (Figures 2A, 4A). The link between the actin cytoskeleton and vacuole structure has been previously studied in *Arabidopsis* root epidermal and egg cells, as well as in Tobacco somatic BY-2 cells, showing that the size and dynamics of vacuoles are F-actin-dependent (Higaki et al., 2006; Kimata et al., 2016; Scheuring et al., 2016).

*A. inaequidens* possesses a highly chalazal polarized central cell nucleus, whose position seems to depend on the actin filaments displayed between the nucleus and the central cell plasma membrane (Figures 2A,B). The positioning of the central cell nucleus by actin filaments was demonstrated by Kawashima and Berger (2015), who disrupted central cell F-actin in *A. thaliana* mature embryo sacs causing a shift of the central cell nucleus from its micropylar to a central position.

After pollination, cortical and perinuclear actin filaments at the central cell gradually changed their configuration, which finally formed an “actin tunnel” mainly composed of several parallel actin cables running from the central cell nucleus to the ovular apparatus at the opposite pole (Figure 3C and Supplementary Video 1). Considering the spatio-temporal establishment of the actin tunnel in *A. inaequidens*, it seems to represent the functional equivalent of the actin cables meshwork of *Arabidopsis* central cell, which show an inward (plasma membrane to nucleus) movement associated with the sperm nuclear migration (Kawashima et al., 2014). Nevertheless, the structure and organization of actin filaments in both systems are distinct. While in *Arabidopsis* central cell F-actin forms a mesh-like structure growing from and attached to the plasma membrane by formins and ROP8, *Agave* actin-tunnel is predominantly formed by parallel actin cables that run along the chalazal-micropylar axis from the central cell nucleus (Figure 3C and Supplementary Video 1). When Ali et al. (2020) inhibited the myosin activity with 50 mM BDM (2, 3-butanedione monoxime) in the *Arabidopsis* central cell, they observed the straightening of the F-actin meshwork, which adopted a similar configuration to those observed in *Agave*.

The exact physiological role of the actin tunnel is intriguing as it is associated with the fertilization process here. Because of the opposite polarity of the tunnel cables (central cell nucleus to the micropylar plasma membrane), it is unlikely they have a homologous function to the mesh-like structure of *Arabidopsis* that seems to escort the sperm nucleus in its transit to the central cell nucleus (Kawashima et al., 2014). Alternatively, *Agave* actin tunnel could have a role in remodeling the central cell vacuole as suggested by the positioning of the tunnel cables, the timing of their development, and the appearance of a trans-vacuolar strand necessary for the transit of the sperm nucleus that fertilizes the central cell (Figures 4, 5A,B).

This trans-vacuolar strand, which traversed the central cell vacuole and was putatively composed of cytoplasm, was observed in Feulgen-stained ovules whose pollen tube had already arrived at one of the synergids (Figures 4, 5A,B and Supplementary Video 2). After plasmogamy, *Agave* sperm nuclei were detected in different regions of the cytoplasmic strand from the egg cell boundary to the border of the central cell nucleus (Figures 5A,B). In *Torenia fournieri*, a similar thick cytoplasmic strand appeared above the ovular apparatus approximately 15 HAP and 5 h after karyogamy of the first sperm nucleus with the egg cell one (Higashiyama et al., 1997). In tobacco, after plasmogamy, some cytoplasmic strands appeared between the sperm nucleus and the central cell nucleus, allowing the former to migrate (Peng et al., 2017). In the presence of cytochalasin B, the cytoplasmic strands were disrupted, and the sperm nucleus migration was prevented (Peng et al., 2017).

Transvacuolar strands also aid in the mobility of polar nuclei, which fuse to form the central cell nucleus. In *Polianthes tuberosa*, another member of the Asparagaceae family, before the migration of the micropylar polar nucleus toward the chalazal end, where the other polar nucleus is located, a thin cytoplasmic strand appears traversing the central vacuole and connecting both polar nuclei (Gonzalez-Gutierrez and Rodriguez-Garay, 2016). Similarly, analysis of the developmental dynamics of *A. thaliana* female gametophyte demonstrated that the migration of polar nuclei occurs through the middle (Susaki et al., 2021) but not along the periphery of the cell (Higaki et al., 2006). Therefore, dynamic changes in the central vacuole could be involved (Susaki et al., 2021).

In agreement with the previous observation, the apparition of the trans-vacuolar strand in *Agave*, and the movement of the sperm nucleus through it was simultaneous with the emergence of a vigorous F-actin mega-cable that extended from the central cell nucleus to the micropylar end (Figures 5C,D). Because of the similar position of the cytoplasmic strand and the F-actin mega-cable within the embryo sac, it is reasonable to hypothesize that the latter fills the space created by the cytoplasmic strand to allow the transit of the sperm nucleus. It is well known that actin filaments are involved in cytoplasmic streaming and that cytoplasmic strands function as transport routes for proteins and organelles (Shimmen and Yokota, 2004). In *A. inaequidens*, the actin mega-cable seems to be the functional equivalent of *Arabidopsis* actin track and aster-like structure associated with the migration of the second sperm nucleus during central cell fertilization (Kawashima et al., 2014). Nevertheless, due to the differences between both structures, the mechanistic implications are also different. While the actin track and the aster-like structure are pleomorphic and do not connect the sites of plasmogamy and karyogamy, the mega-cable establishes a continuous connection between the central cell nucleus and its micropylar end, where most probably the second plasmogamy occurs (Figure 5C). There, the sperm nucleus may be taken and actively transported by the mega-cable until it gets in touch with the central cell nucleus (Figure 5D). In addition to *Agave*, F-actin mega-cables have been observed in other Asparagaceae members such as *Prochnyantes, Yucca, and Manfreda* (González-Gutiérrez et al., 2020).

Although our staining methodology did not allow either higher-resolution or live imaging, our observations suggest that the fertilizing sperm nucleus is wrapped by the F-actin mega-cable, implying that an actin-associated motor protein does not move the sperm nucleus. Instead, the sperm nucleus might be transported together with the mega-cable by a treadmilling mechanism. Although these observations demonstrate a direct association of the actin mega-cable with the second sperm nucleus migration, the sperm nucleus transporting mechanism should be further investigated. Comparative diagrams summarizing the principal differences and similarities between the F-actin structures during the second fertilization of *Agave* and *Arabidopsis* are shown in Figures 5E,F.

Our observations suggest the actin tunnel, but especially the actin-mega cable might be an evolutionary solution in these plant species to the challenge of transporting an immotile sperm nucleus a long distance. Despite the actin tunnel and the mega cable seem to have functional analogs in *Arabidopsis* central cell fertilization (the track and the aster-like structure), their structure and functional scope are clearly different. Why is the fertilizing sperm nucleus in *A. inaequidens* not moved by an aster-like structure? We hypothesize that the differences in F-actin structures developed on each system depend on the distance the sperm nucleus needs to be transported. Actin structures adopt different configurations depending on the distance the cargo needs to be transported in plant cells. Individual or thin actin filaments are associated with short-range cargo targeting, while thicker actin cables are necessary for long-distance transport (Geitmann and Emons, 2000). Because of its thin-cable configuration, an aster-like structure might be more convenient for a short-range movement. Thus, as observed, a robust mega-cable seems a better solution for a long journey.

## Data Availability Statement

The original contributions presented in the study are included in the article/Supplementary Material, further inquiries can be directed to the corresponding author/s.

## Author Contributions

AGG-G performed the experimentation. AGG-G and BR-G designed the experiments. AGG-G, JV, and BR-G analyzed the data and drafted the manuscript. AG-M, JV, and BR-G wrote funding grants. All authors reviewed and approved the final version of the manuscript.

## Conflict of Interest

The authors declare that the research was conducted in the absence of any commercial or financial relationships that could be construed as a potential conflict of interest.

## Publisher’s Note

All claims expressed in this article are solely those of the authors and do not necessarily represent those of their affiliated organizations, or those of the publisher, the editors and the reviewers. Any product that may be evaluated in this article, or claim that may be made by its manufacturer, is not guaranteed or endorsed by the publisher.

## Supplementary Material

Supplementary Video 1 The structure of the actin tunnel in an embryo of *Agave inaequidens* 32–36 HAP. The tunnel is formed by F-actin cables that run from the central cell nucleus at the chalazal end to the micropylar one, and generates a chamber where a vacuole might be contained.

Supplementary Video 2 Feulgen stained *Agave inaequidens* embryo sac (38–42 HAP). After their release from the pollen tube, the two sperm cells stayed together at the chalazal pole of the degenerated receptive synergid cell. Video of a color-coded projection of z-stacked micrographs. Bar = 20 μm.
