# Supplementary material for: An F-Actin Mega-Cable Is Associated With the Migration of the Sperm Nucleus During the Fertilization of the Polarity-Inverted Central Cell of Agave inaequidens
Source: Front Plant Sci. 2021 Nov 24;12:774098. doi: 10.3389/fpls.2021.774098 (PMC8652256; doi:10.3389/fpls.2021.774098)
Supplement: Supplementary file 3 [file Data_Sheet_1.docx]

**Supplementary video 1. The structure of the actin tunnel in an embryo of *Agave inaequidens 32-36 HAP*.** The tunnel is formed by F-actin cables that run from the central cell nucleus at the chalazal end to the micropylar one, and generates a chamber where a vacuole might be contained.

**Supplementary video** **2. Feulgen stained *Agave inaequidens* embryo sac (38-42 HAP).** After their release from the pollen tube, the two sperm cells stayed together at the chalazal pole of the degenerated receptive synergid cell. Video of a color-coded projection of z-stacked micrographs. Bar = 20 μm.
